# Risk of Parkinson Disease Among Adults With vs Without Posttraumatic Stress Disorder

**DOI:** 10.1001/jamanetworkopen.2022.25445

**Published:** 2022-08-04

**Authors:** Yael Barer, Gabriel Chodick, Nurit Glaser Chodick, Tanya Gurevich

**Affiliations:** 1 Maccabitech, Maccabi Institute for Research and Innovation, Maccabi Healthcare Services, Tel Aviv, Israel; 2 Sackler School of Medicine, Tel Aviv University, Tel Aviv, Israel; 3 Bob Shapell School of Social Work, Tel Aviv University, Tel Aviv, Israel; 4 Tel Aviv Sourasky Medical Center, Tel Aviv, Israel; 5 Sagol School of Neuroscience, Tel Aviv University, Tel Aviv, Israel

## Abstract

**Importance:**

Posttraumatic stress disorder (PTSD) in elderly men may be associated with increased risk of Parkinson disease (PD); thus, this group of patients needs to be monitored closely for timely, customized treatment.

**Objective:**

To evaluate the risk of PD in patients with PTSD compared with patients without PTSD.

**Design, Setting, and Participants:**

This population-based, retrospective, cohort study used data from Maccabi Health Care Services (MHS), the second largest health plan in Israel, spanning from 2000 to 2019. Participants included MHS members born before 1970 who received a diagnosis of PTSD in 2000 to 2015. Patients with PTSD who had PD before their first diagnosis were excluded. Data analysis was performed from February to June 2022.

**Exposures:**

Incident PTSD was denoted by at least 1 diagnosis (1) given by psychiatrists, psychologists, or neurologists; (2) hospital discharge diagnosis; or (3) registered as a chronic diagnosis (defined as such by the primary care physician). The index date was defined as first diagnosis for the patients with PTSD and for the corresponding patients without PTSD.

**Main Outcomes and Measures:**

PD incident cases up to 2019 were ascertained by idiopathic PD diagnosis (1) given by a neurologist, (2) extracted from a hospital discharge report, or (3) registered as a chronic diagnosis. Patients with PD-like syndromes documentation after the last mention of PD were excluded.

**Results:**

Of 8342 eligible patients, 8336 (99.9%) were matched to nonexposed patients in a 1:1 ratio by birth year and sex; 4303 patients (51.6%) were male, and the mean (SD) age at index was 55.8 (13.2) years. Patients with PTSD had a 1.48-fold (95% CI, 1.10-1.99) excess risk for PD, compared with patients without PTSD. An elevated risk of PD (hazard ratio, 1.95; 95% CI, 1.16-3.28) was recorded among men receiving a diagnosis of PTSD at age 72 years or older.

**Conclusions and Relevance:**

These findings suggest that elderly men who receive a diagnosis of PTSD are at an increased risk of PD. Further studies are needed to corroborate these findings and to further assess the association of stress with PD risk.

## Key Points

**Question:**

Is a history of posttraumatic stress disorder (PTSD) associated with an increased risk of Parkinson disease (PD)?

**Findings:**

In this cohort study of 8336 patients with PTSD matched with 8336 patients without PTSD, patients with PTSD had an increased risk of PD compared with those without PTSD. Risks were higher among men older than 72 years.

**Meaning:**

These findings suggest that older men with a history of PTSD should be monitored for PD.

## Teaser

This cohort study evaluates the comparative risk of Parkinson disease in patients with and without posttraumatic stress disorder.

## Introduction

Parkinson disease (PD) is the second most common neurodegenerative disease, after Alzheimer disease, with a lifetime prevalence of approximately 1.5%.^1,2^ PD is manifested by the loss of dopamine-producing neurons in the substantia nigra, resulting in various motor and nonmotor symptoms.^3^ PD risk is associated with multiple environmental, behavioral, and biological^4^ risk factors.

Posttraumatic stress disorder (PTSD) is a mental disorder that occurs as a result of an exposure to a traumatic event.^5^ Dopamine is implicated in the regulation of fear conditioning and anxiety.^6^ In individuals with PTSD, there is a genetic component associated with dopamine metabolism that governs whether an individual develops PTSD, as well as what symptoms they may display.^6^ The prevalence of traumatic exposure worldwide is 70.4% (range, 28.6%-84.6%), and Israel ranks high, with a prevalence of 74.8%.^7^ Consequently, it has been estimated that 9.4% of Israel’s adult population has PTSD,^8^ more than twice the global prevalence of 3.9%.^9^

PTSD, along with trauma exposure, is associated with increased incidence of various diseases,^10,11,12,13^ including assorted dementias and Alzheimer disease.^14,15,16^ The association between stress (including PTSD) and PD has been hypothesized since the 1980s,^17,18^ but longitudinal cohort studies using systematic data collection are scarce. A cohort study^19^ in Taiwan found a 3.46-fold risk for PD in late life compare with patients without PTSD. Recently, a case-control study^20^ has shown that PTSD is associated with higher odds of PD (odds ratio, 2.7) in veterans in the US. However, that study population was almost 99% male and assessed prevalent PD; thus, the measured PD risk might be biased and overestimated. In the current study, we evaluated the association between PTSD and incident PD, using data from the second largest health plan in Israel over the course of a 20-year follow-up period.

## Methods

### Study Design and Population

This retrospective cohort study was performed using data from the Maccabi Health Care Services (MHS) automatically computerized clinical database. MHS is a nationwide health plan (payer-provider) representing approximately one-quarter of the population in Israel (approximately 2.6 million people) with less than 1% per year moving out. The National Health Insurance Law passed in Israel in 1995 mandates that all legal residents be required to enroll in 1 of 4 not-for-profit health plans and guarantees free choice among them.^21^ The study was conducted in accordance with the International Council for Harmonisation of Technical Requirements for Pharmaceuticals for Human Use Guideline for Good Clinical Practice, Good Epidemiology Practices^22^ and Declaration of Helsinki, Ethical Principles for Medical Research Involving Human Subjects.^23^ A waiver of consent was approved by the Maccabi ethics committee because this retrospective database study was performed using data that were automatically collected in the MHS databases as part of routine data collection in a large health maintenance organization. All data were anonymous. This study followed the Strengthening the Reporting of Observational Studies in Epidemiology (STROBE) reporting guideline for cohort studies.

MHS members born before 1970 with a PTSD diagnosis (*International Classification of Diseases, Ninth Edition* [*ICD-9*] code 309.81) (1) given by selected specialists (ie, psychiatrists, psychologists, or neurologists), (2) extracted from a hospital discharge report, or (3) documented as a chronic diagnosis (defined as such by the primary physician), between January 1, 2000, and December 31, 2015, were identified. We required at least 1 year of continuous membership in MHS before the first PTSD diagnosis to ensure that cases were incident. Patients with PD diagnosed before the first PTSD diagnosis were excluded from the analysis. All eligible patients with PTSD were randomly matched to MHS members without PTSD with at least 1 year of membership on a 1:1 basis by exact birth year and sex. The index date was defined as the date of the first PTSD diagnosis. Patients without PTSD were given the index date of the their respective matched patient with PTSD. All patients without PTSD were PD free at the index date.

### PD Ascertainment

PD cases until the end of 2019 were ascertained by idiopathic PD diagnosis (*ICD-9* codes 332 and 332.0) (1) given by a neurologist, (2) extracted from a hospital discharge report, or (3) documented as a chronic diagnosis. Patients whose last idiopathic PD diagnosis was followed with 1 of the following diagnoses given by the previous mentioned experts were excluded: progressive supranuclear palsy (*ICD-9* code 333.0), multiple system atrophy (*ICD-9* code 333.0), drug-induced parkinsonism (*ICD-9* code 332.1), essential tremor (*ICD-9* code 333.1), or tardive dyskinesia (*ICD-9* codes 333.82 and 333.85).

### Variables and Measurements

Patients’ demographics at index date included age, sex, smoking status (current, past, and never), body mass index (calculated as weight in kilograms divided by height in meters squared), and socioeconomic status (SES). SES was based on a score ranked with 1 (lowest) to 10 (highest) derived for commercial purposes by Points Location Intelligence using geographic information systems and data such as expenditures related to retail chains, credit cards, and housing. This score is highly correlated with SES measured by the Israel Central Bureau of Statistics.^24,25^ SES was categorized according to population tertiles as low (SES score 1-4), medium (SES score 5-6), and high (SES score 7-10). Data regarding Holocaust survivors and survivors of terror attacks were assessed using indication given by the Israeli social security. Patients’ comorbidities at the index date were also extracted. Whenever possible, MHS-validated registries were used: cardiovascular disease registry^26^ (including both cardiovascular disease and stroke data), diabetes,^27^ hypertension,^28^ chronic kidney disease,^29^ and cancer.^30^ Additional comorbidities (ie, depression, migraine, epilepsy, and traumatic brain injury) were defined as present when at least 3 diagnoses were documented, with at least first mention before the index date. End of follow-up was defined as the earlier of (1) death date, (2) PD first diagnosis, (3) leaving MHS date, or (4) end of study on December 31, 2019.

### Statistical Analysis

 Data analysis was performed from February to June 2022. Descriptive statistics are presented using frequencies and proportions for categorical variables and mean values with SD or medians with IQRs as appropriate. Standard mean differences were calculated to assess the absolute effect size for each baseline comparison. A standardized mean difference less than 0.1 was taken to indicate a negligible difference in the mean or prevalence of a covariate between exposure groups.^31^ Time to incident PD in patients with and without PTSD was calculated using Kaplan-Meier curves and compared using the log-rank test. The proportionality assumption was assessed using Schoenfeld residuals test and verified both by a nonsignificant *P* value (*P* = .19; global test, *P* = .74) and by visual assessment of the residual plot (data not shown). Cox proportional hazards regression was used to estimate hazard ratios (HRs) and 95% CIs of PD among patients with PTSD compared with those without PTSD. Multivariable Cox proportional hazards was performed to adjust for suspected confounders measured at baseline (variable with standard mean difference >0.1 at baseline). Analysis stratification was performed by age at index date (according to the median age at index date of the PD cases, 72 years) and sex. A few sensitivity analyses were performed. First, we excluded patients (and their matched controls) with an index date before 2004, to further ensure incident cases of PTSD. Second, we excluded patients who received a diagnosis of PD in the first year after the PTSD index date (and their matched controls) to ensure that PTSD precede the development of PD. Third, we excluded patients with PD (and their matched controls) who had at least 1 purchase of antipsychotic medication in the year before their first PD diagnosis because of the motor effects of antipsychotic medications. Fourth, to address the possibility of ascertainment bias (which might be introduced when intense surveillance or screening for outcomes among individuals with a history of PTSD is greater than among unexposed individuals), we examined the association between PTSD and negative control outcomes, including neurological diseases (eg, glioblastoma multiform amyotrophic lateral sclerosis, Creutzfeldt-Jakob disease, and Huntington disease) that may share the same potential ascertainment bias but are not caused by PTSD. The presence of these outcomes was assessed after the index date using the same criteria as for PD. We excluded patients with an event before the index date. To maintain balanced cofounders, each excluded case was excluded with their matched control. Fifth, we also restricted the analyses to those with no brain injury history to assess the possibility of confounding due to brain injury. In addition, we performed a sensitivity analysis replacing the categorical SES with the original continuous variable.

Two-tailed *P* < .05 was considered significant. All analyses were performed in SPSS statistical software version 27 (IBM-SPSS). Figures were created using ggplot2, survival, and survminer packages in R statistical software version 4.1.1 (R Project for Statistical Computing).

## Results

### Patients' Characteristics

**Figure 1.**
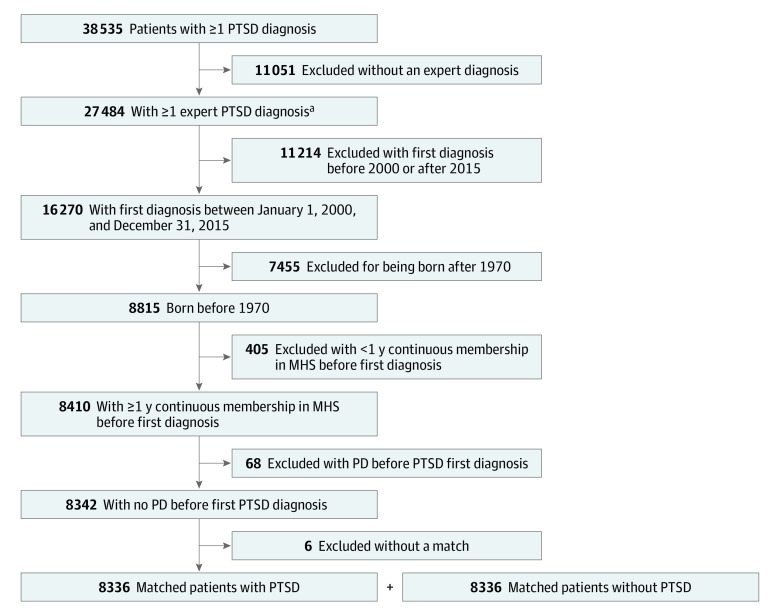
Patient Enrollment Flowchart MHS indicates Maccabi Health Care Services; PD, Parkinson disease; PTSD, posttraumatic stress disorder. ^a^Expert diagnosis is defined as a diagnosis (1) given by selected specialists (ie, psychiatrists, psychologists, or neurologists), (2) in a hospital discharge report, or (3) with a chronic PTSD diagnosis (defined as such by the primary physician).

We identified and matched 8336 of 8342 eligible patients (99.9%) with PTSD (4303 men [51.6%]; mean [SD] age at index date, 55.8 [13.2] years) (Figure 1). The mean (SD) follow-up time after the index date was 10.2 (4.8) years for patients without PTSD and 10.4 (4.7) years for patients with PTSD. Among the patients without PTSD, 508 (6.1%) were lost to follow-up (183 [36.0%] transferred to another health plan and 307 [60.%] moved abroad). Among patients with PTSD, 298 (3.6%) were lost to follow-up (198 [66.4%] transferred to another health plan and 89 [29.9%] moved abroad). The rest of the study participants who were lost to follow-up (18 patients without PTSD [3.5%] and 11 patients with PTSD [3.7%]) stopped MHS membership for other reasons.

Patients with PTSD were more likely to be of low-to-medium SES, current smokers (1255 patients [15.1%] vs 945 patients [11.3%]), Holocaust survivors (904 patients [10.8%] vs 568 patients [6.8%]), and survivors of terror attacks (235 patients [2.8%] vs 8 patients [0.1%]) compared with patients without PTSD (Table 1). Patients with PTSD were also more likely to have various comorbidities at baseline, including hypertension (3003 patients [36.0%] vs 2602 patients [31.2%]), depression (518 patients [6.2%] vs 97 patients [1.2%]), migraine (570 patients [6.8%] vs 368 patients [4.4%]), and brain injury (843 patients [10.1%] vs 84 patients [1.0%]), compared with patients without PTSD (Table 1).

**Table 1.** Baseline Characteristics for Patients With PTSD and Matched Patients Without PTSD

Characteristic	Patients, No. (%)		SMD^a^
	Without PTSD (n = 8336)	With PTSD (n = 8336)	
Age at index, y			
Mean (SD)	55.8 (13.2)	55.8 (13.2)	<.001
<72	7025 (84.3)	7025 (84.3)	<.001
≥72	1311 (15.7)	1311 (15.7)	
Sex			
Male	4303 (51.6)	4303 (51.6)	<.001
Female	4033 (48.4)	4033 (48.4)	
Socioeconomic status^b^			
Low	1442 (17.3)	1602 (19.2)	.14
Medium	2828 (33.9)	3246 (38.9)	
High	4006 (48.1)	3449 (41.4)	
Missing	60 (0.7)	39 (0.5)	
Mean (SD)	6.4 (1.9)	6.1 (1.8)	.17
Smoking status			
Current	945 (11.3)	1255 (15.1)	.11
Past	223 (2.7)	229 (2.7)	
Never	4799 (57.6)	4525 (54.3)	
Unknown	2369 (28.4)	2327 (27.9)	
Body mass index, mean (SD)^c^	28.4 (5.4)	28.4 (5.2)	.002
Holocaust survivor	568 (6.8)	904 (10.8)	.14
Birth year 1933-1945^d^	432 (31.7)	668 (49.0)	.36
Survivor of terror attack	8 (0.1)	235 (2.8)	.23
Underlying conditions			
Cardiovascular disease	731 (8.8)	954 (11.4)	.09
Stroke	173 (2.1)	233 (2.8)	.05
Diabetes	989 (11.9)	1230 (14.8)	.09
Hypertension	2602 (31.2)	3003 (36.0)	.10
Cancer	658 (7.9)	685 (8.2)	.01
Chronic kidney disease	1547 (18.6)	1642 (19.7)	.03
Depression	97 (1.2)	518 (6.2)	.27
Migraine	368 (4.4)	570 (6.8)	.11
Epilepsy	44 (0.5)	86 (1.0)	.06
Brain injury	84 (1.0)	843 (10.1)	.41
Follow-up time, mean (SD), y	10.2 (4.8)	10.4 (4.6)	.04

Abbreviations: PTSD, posttraumatic stress disorder; SMD, standard mean difference.

^a^ SMD is an absolute effect size. An SMD less than 0.1 was taken to indicate a negligible difference in the mean or prevalence of a covariate between groups.

^b^ Socioeconomic status was based on a score ranked from 1 (lowest) to 10 (highest) using geographic information and data such as expenditures related to retail chains, credit cards, and housing and was categorized according to population tertiles as low (score 1-4), medium (score 5-6), and high (score 7-10).

^c^ Body mass index is calculated as weight in kilograms divided by height in meters squared.

^d^ Analysis includes only patients born between 1933 and 1945 (World War II period).

### Risk of PD

**Figure 2.**
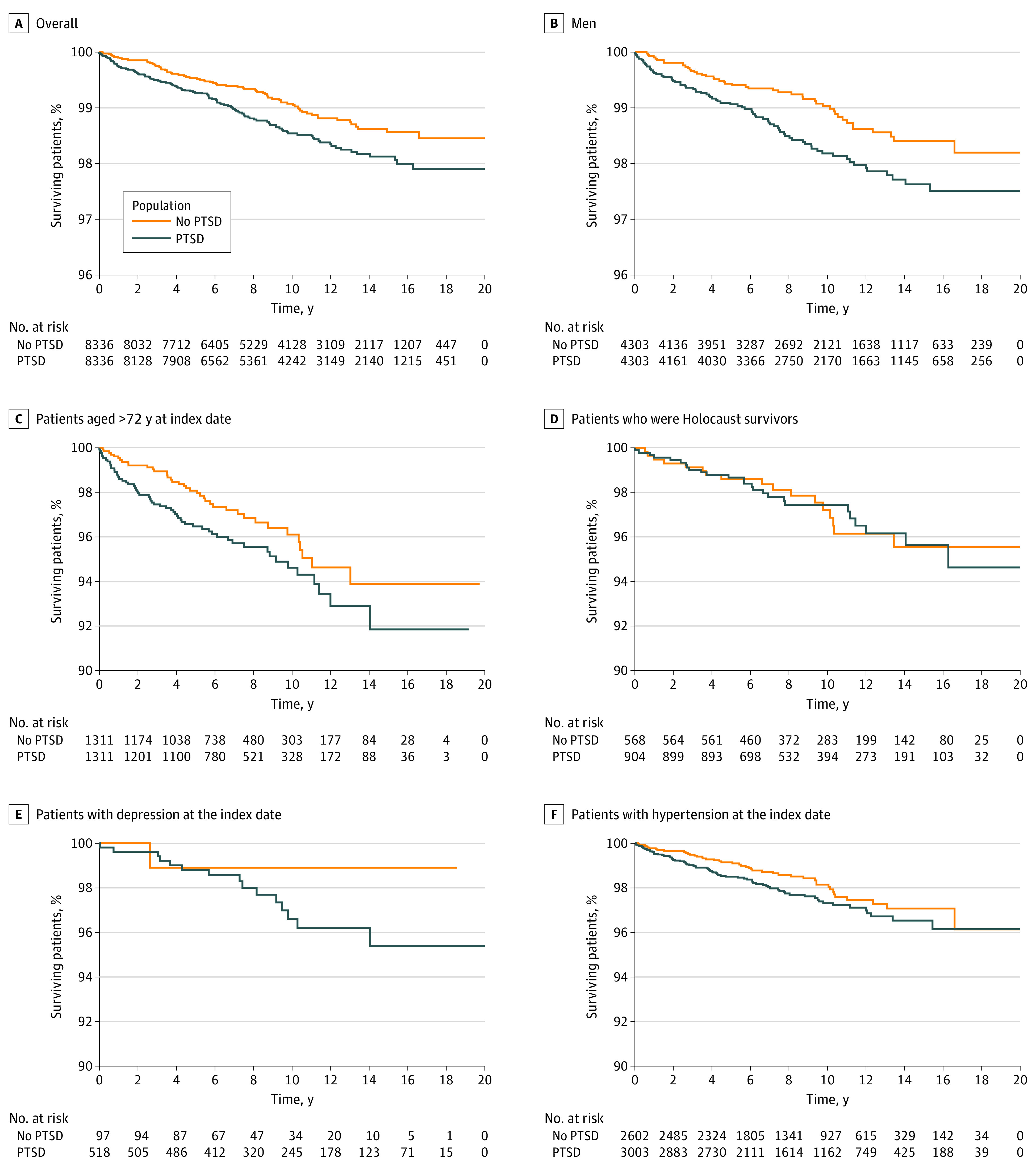
Association of History of Posttraumatic Stress Disorder (PTSD) With Parkinson Disease Graphs show data for entire cohort (A), men only (B), patients older than 72 years at the index date (C), Holocaust survivors (D), patients with depression at the index date (E), and patients with hypertension at the index date (F).

PD developed in 117 patients with PTSD (1.4%) and 79 patients without PTSD (0.9%). Figure 2 presents survival curves of patients with PTSD vs patients without PTSD for all study population and by different baseline confounders. Patients with PTSD had an adjusted HR of 1.48 (95% CI, 1.10-1.99) for developing PD, compared with patients without PTSD. Stratified analysis by age and sex revealed an adjusted HR of 1.95 (95% CI, 1.16-3.28) for developing PD in male patients with PTSD who received a diagnosis of PTSD at the age of 72 years or older (Table 2).

**Table 2.** HRs and 95% CIs for PD in Patients With PTSD Compared With Patients Without PTSD

Variable	Patients with PD, No./total No.		Unadjusted		Adjusted^a^	
	Without PTSD	With PTSD	HR (95% CI)	*P* value	HR (95% CI)	*P* value
Main analysis overall	79/8336	117/8336	1.45 (1.09-1.93)	.01	1.48 (1.10-1.99)	.01
Stratified by age and sex						
Male						
<72 y	22/3651	33/3651	1.48 (0.86-2.53)	.16	1.57 (0.89-2.76)	.12
≥72 y	24/652	42/652	1.72 (1.04-2.83)	.04	1.95 (1.16-3.28)	.01
Female						
<72 y	18/3374	25/3374	1.37 (0.75-2.50)	.31	1.40 (0.75-2.63)	.29
≥72 y	15/659	17/659	1.07 (0.53-2.15)	.85	1.11 (0.53-2.33)	.78
Sensitivity analysis						
PTSD exposure after 2003, overall	64/6787	90/6787	1.38 (1.00-1.90)	.05	1.43 (1.02-1.99)	.04
PD after first year, overall	70/8308	97/8308	1.36 (0.99-1.84)	.05	1.33 (0.96-1.83)	.08
Exclude PD cases with antipsychotic use 1 y before PD, overall	71/8308	97/8308	1.34 (0.99-1.82)	.06	1.40 (1.03-1.91)	.03
Restriction for patients with no indication of brain injury history	79/8252	111/7493	1.54 (1.15-2.05)	.004	1.47 (1.09-1.97)	.01
Socioeconomic status as a continuous variable	79/8276	116/8297	1.44 (1.08-1.92)	.01	1.50 (1.12-2.00)	.006

Abbreviations: HR, hazard ratio; PD, Parkinson disease; PTSD, posttraumatic stress disorder.

^a^ Adjusted for age at index, sex, socioeconomic status, smoking status, Holocaust survivor and survivor of terror attack, hypertension, depression, migraine, and traumatic brain injury.

Sensitivity analyses were conducted. The results were not materially different after excluding members with an index date before 2004 (overall adjusted HR, 1.43; 95% CI, 1.02-1.99) (Table 2) (data are shown stratified by age and sex in eTable 1 in the Supplement). When we excluded patients with less than 1 year between the index date and the first diagnosis of PD, there was no association between PD and PTSD (Table 2 and eTable 1 in the Supplement). After excluding PD cases with antipsychotic medication use in the year before PD diagnosis, the results were similar to those observed in the main analysis (overall adjusted HR, 1.40; 95% CI, 1.03-1.91) (Table 2 and eTable 1 in the Supplement). A negative control outcomes analysis found no difference in the risk among patients with and without PTSD (event rate in both groups, 0.2%; HR, 1.00; 95% CI, 0.50-1.90; *P* > .99) (eTables 2 and 3 in the Supplement). Restriction of the main analysis to those with no indication of brain injury history showed estimated risk similar to that found by the main analysis (overall adjusted HR, 1.54; 95% CI, 1.15-2.05) (eFigure in the Supplement). The final sensitivity analysis using SES as a continuous variable produced results almost identical to those in the main analysis (overall adjusted HR, 1.50; 95% CI, 1.13-2.00). Stratified analyses by age and sex of sensitivity analyses are presented in Table 2 and in eTable 1 in the Supplement.

## Discussion

The current retrospective cohort study spanning more than 20 years of follow-up found that incident PTSD was associated with an increased risk for PD in male patients, particularly among those aged 72 years and older. These findings are consistent with the results of a recent study by White et al^20^ among male veterans in the US showing a 2.7-fold excess risk for PD. The association between PTSD and PD among elderly male patients in our analysis was robust and not affected by adjustments for depression and traumatic lifetime events, such as the Holocaust and experiencing terror attacks. In addition, the results of the main analysis were solid across several sensitivity analyses, including restriction for patients with no history of brain injury. Although in our analysis the association between PTSD and PD in female patients was not significant, a 3.46-fold increased risk for PD in patients with history of PTSD compared with those without PTSD was previously reported in a predominantly female cohort.^19^

Over the last few decades, emotional and physical stress have been linked to PD risk.^18^ Furthermore, there might be a link between the duration of PTSD and PTSD-related neurodegeneration.^32^ Moreover, sleep disturbance, a common symptom of PTSD manifested as nightmares and difficulty in falling asleep and maintaining sleep, may disrupt the balance of oxidants and antioxidants toward a more oxidative environment.^32^ Nonetheless, the causal association between stress or anxiety and PD has not been fully established. A recent study^33^ has shown that adjustment disorder, an emotional or behavioral reaction to a stressful event, was associated with increased risk of PD. Another study^34^ has shown that anxious personality increases the risk of PD in men by 2-fold and by 1.76-fold in people aged 50 to 69 years at the time they took the Minnesota Multiphasic personality inventor test. Moreover, depression and anxiety are well recognized prodromal symptoms of PD.^35^ PTSD may present the extreme manifestation of less-acute disorders, such as anxiety, depression, or stressful existence. The fact that PTSD in men older than 72 years was associated with increased risk of developing PD later in life in our study may indicate that PTSD in older patients may actually be a prodromal symptom of PD, especially in the light of the fact that *PARK2*, a PD gene involved in dopamine regulation, is associated with PTSD in men, along with other novel genes and noncoding RNAs.^36^

### Limitations and Strengths

The current analysis has several limitations. First, data regarding the type of trauma and PTSD severity were not available for extraction; thus, we were unable to further investigate the association of PTSD with PD. Second, sensitivity analysis excluding PD cases in the first year after PTSD diagnosis showed an attenuation of risk estimates. It is well known that PD has a long prodromal phase^37^; thus, exposure to risk factors would have accrued at least 10 to 15 years before PD diagnosis (ie, the appearance of distinct motor symptoms). However, it is also known that PTSD diagnosis is not necessarily documented at first appearance of symptoms because of (1) strict diagnosis criteria when subsyndromal symptoms accumulate with time until being sufficient for diagnosis; (2) early PTSD episodes being unrecognized as such, undisclosed, or forgotten; or (3) remanifestation of PTSD in older age as a result of stressors such as loneliness and physical illness.^38^ In addition, in 2000 the Community Rehabilitation of Persons with Mental Health Disability law^39^ was accepted in Israel, making patients with PTSD (among others) entitled to a package of social services. This reform might have encouraged previously symptomatic undiagnosed patients to be officially diagnosed at more late stage of the disorder. Third, despite a known female predominance of PTSD,^40^ this analysis has found similar proportions of PTSD in men and women. In Israel, there is a higher proportion of men rather than women in combat units in the Israeli defense forces. In men, combat is the second leading cause of PTSD, with approximately 40% of the men experiencing combat developing PTSD.^5^ In the current analysis, we included Israeli citizens born before 1970, a population who was exposed to several regional wars either in the army (age 18 years) or in the reserve services (age ≥21 years).

Additional limitations of this study are those inherent in the use of database analysis, including the use of *ICD-9–Clinical Modification* codes to identify patients with PTSD. It is possible that some individuals are misclassified as having as PTSD or trauma brain injury that share a similar clinical presentation. This is indicated by the high proportion of brain injury among patients with PTSD. In addition, unlike *International Classification of Diseases, 11th Revision*, *ICD-9* provides no distinction between PTSD and complex PTSD, which is characterized by additional clusters that reflect disturbances in self-organization, such as affective dysregulation, negative self-concept, and disturbances in relationships.^41^ Complex PTSD was associated with sustained, repeated, or multiple forms of traumatic exposure. The specific relationship between complex PTSD and PD could not have been investigated in the current study and warrants future research.

This study has several strengths. First, to best of our knowledge, this is one of the first large-scale longitudinal cohort studies examining the risk of PD in patients with PTSD. Second, owing to the long duration of data availability, we were able to include a large number of patients with PTSD and allow for at least 5 years of postexposure follow-up. Third, we were able to minimize the concern of ascertainment bias by showing no difference in other neurological diagnosis between those with and without PTSD.

## Conclusions

Our study showed that PTSD diagnosed in men over the age of 72 years is associated with an increased risk of developing PD later in life. Thus, PTSD may be considered to be a factor associated with increased risk for PD or PD prodromal symptom, which warrants further investigation. In addition, further research is needed to ascertain whether treatment for PTSD and stress can reduce the risk or rate of progression for PD.

## Notes

Supplement. **eTable 1.** Hazard Ratios and 95% Confidence Intervals (95% CI) for PD in PwPTSD Compared to Patients Without PTSD **eTable 2.** Negative Control Outcomes **eTable 3.** Hazard Ratios and 95% Confidence Intervals (95% CI) for the Negative Control Outcome in Patients With PTSD Compared to Patients Without PTSD **eFigure.** Relationship Between History of PTSD and Parkinson's Disease Restricted for Those With No Indication of Brain Injury History
