# Supplementary material for: Risk of Parkinson Disease Among Adults With vs Without Posttraumatic Stress Disorder
Source: JAMA Netw Open. 2022 Aug 4;5(8):e2225445. doi: 10.1001/jamanetworkopen.2022.25445 (PMC9353613; doi:10.1001/jamanetworkopen.2022.25445)
Supplement: Supplement. — eTable 1. Hazard Ratios and 95% Confidence Intervals (95% CI) for PD in PwPTSD Compared to Patients Without PTSD eTable 2. Negative Control Outcomes eTable 3. Hazard Ratios and 95% Confidence Intervals (95% CI) for the Negative Control Outcome in Patients With PTSD Compared to Patients Without PTSD eFigure. Relationship Between History of PTSD and Parkinson's Disease Restricted for Those With No Indication of Brain Injury History [file jamanetwopen-e2225445-s001.pdf]

Supplemental Online Content

Barer Y, Chodick G, Glaser Chodick N, Gurevich T. Risk of Parkinson disease among adults with vs without posttraumatic stress disorder. *JAMA Netw Open*. 2022;5(8):e2225445. doi:10.1001/jamanetworkopen.2022.25445

eTable 1. Negative Control Outcomes

eTable 2. Hazard Ratios and 95% Confidence Intervals (95% CI) for the Negative Control Outcome in Patients With PTSD Compared to Patients Without PTSD

eTable 3. Hazard Ratios and 95% Confidence Intervals (95% CI) for PD in PwPTSD Compared to Patients Without PTSD

eFigure. Relationship Between History of PTSD and Parkinson's Disease Restricted for Those With No Indication of Brain Injury History

This supplemental material has been provided by the authors to give readers additional information about their work.

© 2022 Barer Y et al. *JAMA Network Open*.

eTable 1: Negative Control Outcomes

Negative control outcome	no PTSD (n=8325)<br>n = 20	PTSD (n=8325)<br>n = 18
GBM	14 (70.0%)	13 (65.0%)
ALS	2 (10.0%)	3 (15.0%)
CJD	3 (15.0%)	1 (5.0%)
HD	1 (5.0%)	1 (5.0%)

GBM: glioblastoma multiform, ALS: amyotrophic lateral sclerosis, CJD: Creutzfeldt-Jakob Disease, HD: Huntington's disease, PTSD: posttraumatic stress disorder

eTable 2: Hazard Ratios and 95% Confidence Intervals (95% CI) for the Negative Control Outcome in Patients With PTSD Compared to Patients Without PTSD

No PTSD	PTSD	Unadjusted		Adjusted	
n outcomes / N total	n outcomes / N total	HR (95%CI)	p-value	HR (95%CI)	p-value
20 / 8325	18 / 8325	0.88 (0.47-1.67)	.70	1.00 (0.53-1.91)	.99

[a]Adjusted for: age at index, sex, SES, smoking status, holocaust survivor and victim of terror, hypertension, depression, migraine and traumatic brain injury.
PTSD: posttraumatic stress disorder, PD: Parkinson's disease, HR: hazard ration, CI: confidence interval, SES: socioeconomic status

© 2022 Barer Y et al. *JAMA Network Open*.

eTable 3: Hazard Ratios and 95% Confidence Intervals (95% CI) for PD in PwPTSD Compared to Patients Without PTSD

			No PTSD	PTSD	Unadjusted		Adjusted [a]	
			n PD / N total	n PD / N total	HR (95%CI)	p-value	HR (95%CI)	p-value
**Main analysis**	Overall		79 / 8336	117 / 8336	1.45 (1.1-1.9)	0.01	1.48 (1.1-2)	.01
	Stratified by age and sex	Male, <72	22 / 3651	33 / 3651	1.48 (0.9-2.5)	.16	1.57 (0.9-2.8)	.12
		Male, ≥72	24 / 652	42 / 652	1.72 (1.04-2.8)	.04	1.95 (1.2-3.3)	.01
		Female, <72	18 / 3374	25 / 3374	1.37 (0.7-2.5)	.31	1.40 (0.7-2.6)	.29
		Female, ≥72	15 / 659	17 / 659	1.07 (0.5-2.1)	.85	1.11 (0.5-2.3)	.78
**Sensitivity analysis.** PTSD exposure >2003	Overall		64 / 6787	90 / 6787	1.38 (1.02-1.9)	.05	1.43 (1-2)	.04
	Stratified by age and sex	Male, <72	13 / 2873	24 / 2873	1.83 (0.9-3.6)	.08	1.95 (1-3.9)	.06
		Male, ≥72	22 / 584	36 / 584	1.61 (0.9-2.7)	.08	1.88 (1.1-3.3)	.03
		Female, <72	16 / 2730	16 / 2730	0.97 (0.5-1.9)	.94	1.09 (0.5-2.2)	.82
		Female, ≥72	13 / 600	14 / 600	1.02 (0.5-2.2)	.95	1.09 (0.5-2.4)	.84
**Sensitivity analysis.** At least one year between PTSD and PD	Overall		70 / 8308	97 / 8308	1.36 (1-1.8)	.05	1.33 (1-1.8)	.08
	Stratified by age and sex	Male, <72	21 / 3647	30 / 3647	1.4 (0.8-2.5)	.23	1.49 (0.8-2.7)	.18
		Male, ≥72	20 / 638	31 / 638	1.42 (0.8-2.5)	.22	1.49 (0.8-2.7)	.18
		Female, <72	17 / 3372	24 / 3372	1.39 (0.7-2.6)	.30	1.48 (0.8-2.8)	.24
		Female, ≥72	12 / 651	12 / 651	0.93 (0.4-2.1)	.86	0.96 (0.4-2.2)	.92
**Sensitivity analysis.** Exclude PD cases with antipsychotic medication one year prior PD	Overall		71 / 8308	97 / 8308	1.34 (1-1.8)	.06	1.4 (1.03-1.9)	.03
	Stratified by age and sex	Male, <72	22 / 3646	28 / 3646	1.25 (0.7-2.2)	.43	1.32 (0.7-2.4)	.35
		Male, ≥72	22 / 646	38 / 646	1.69 (1-2.9)	.05	1.91 (1.1-3.3)	.02
		Female, <72	14 / 3365	20 / 3365	1.4 (0.7-2.8)	.33	1.51 (0.7-3.1)	.26
		Female, ≥72	13 / 651	11 / 651	0.79 (0.4-1.8)	.57	0.97 (0.4-2.2)	.95
**Sensitivity analysis.** SES as a continuous variable	Overall		79 / 8276	116 / 8297	1.44 (1.1-1.9)	0.013	1.5 (1.1-2)	0.006
	Stratified by age and sex	Male, <72	22 / 3633	33 / 3635	1.47 (0.9-2.5)	0.158	1.61 (0.9-2.8)	0.088
		Male, >=72	24 / 646	42 / 651	1.7 (1-2.8)	0.038	1.88 (1.1-3.1)	0.016
		Female, <72	18 / 3349	25 / 3360	1.36 (0.7-2.5)	0.317	1.46 (0.8-2.7)	0.224
		Female, >=72	15 / 648	16 / 651	1 (0.5-2)	0.999	1.05 (0.5-2.1)	0.901

[a]Adjusted for: age at index, sex, SES, smoking status, holocaust survivor and victim of terror, hypertension, depression, migraine and traumatic brain injury.

PTSD: posttraumatic stress disorder, PD: Parkinson's disease, HR: hazard ration, CI: confidence interval, SES: socioeconomic status

© 2022 Barer Y et al. *JAMA Network Open*.

eFigure: Relationship Between History of PTSD and Parkinson's Disease Restricted for Those With No Indication of Brain Injury History

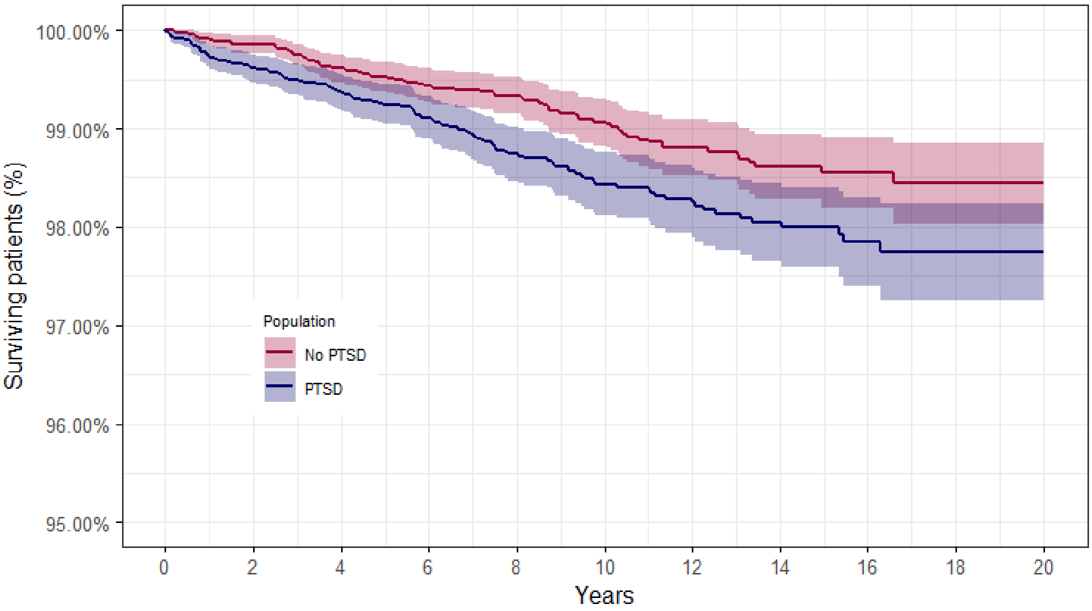

Number at risk

No PTSD	8252	7949	7633	6347	5191	4098	3083	2104	1202	446	0
PTSD	7493	7295	7090	5852	4736	3729	2753	1878	1063	384	0

Kaplan Meier survival functions by PTSD history status are shown for Parkinson's diseases incident diagnoses.

© 2022 Barer Y et al. *JAMA Network Open*.
